# Consultations in general practice related to a tick bite episode in mainland France in 2023–2024

**DOI:** 10.1186/s12889-026-27498-8

**Published:** 2026-04-29

**Authors:** Camille Bonnet, Titouan Launay, Camille Coustaury, Alexandra Septfons, Jonas Durand, Pascale Frey-Klett, Sandrine Warion, Clémence Galon, Sara Moutailler, Julie Sevila, Raphaëlle Métras, Marion Debin, Pierre-Yves Boëlle, Benoît Jaulhac, Julie Figoni, Thierry Blanchon, Alessandra Falchi

**Affiliations:** 1 Sorbonne Université, INSERM, Institut Pierre Louis d’Épidémiologie et de Santé Publique, IPLESP, Paris, F75012 France; 2 Santé publique France, Saint-Maurice, France; 3 ANSES, INRAE, Ecole Nationale Vétérinaire d’Alfort, UMR BIPAR, Laboratoire de Santé Animale, Maisons-Alfort, France; 4 Unité Infectiologie Eco-épidémiologie Risque Faune Sauvage, ANSES, Laboratoire de la rage et de la faune sauvage de Nancy, Malzéville, France; 5 Laboratoire de Santé Animale, ANSES, INRAE, Ecole Nationale Vétérinaire d’Alfort, UMR BIPAR, Maisons-Alfort, France; 6 Unité des Virus Émergents (UVE), Università di Corsica, IRBA, Aix-Marseille Université, IRD 190, Inserm, 1207 France; 7 CNR des Borrelia et Institut de Bactériologie, Fédération de Médecine Translationnelle de Strasbourg, Université de Strasbourg, CHRU Strasbourg, UR3073-PHAVI, 3 rue Koeberlé, Strasbourg, 67000 France

**Keywords:** Tick Bites, Tick-Borne Diseases, General Practice, France, Incidence

## Abstract

**Background:**

The emergence and spread of TBDs present major challenges. Exposure to tick bites in mainland France is mainly documented through local studies or citizen science projects. According to the 2024 French national survey, about 15% bitten individuals consult a healthcare professional. National-level data from general practice are needed to better describe tick bite epidemiology.

**Main objective:**

To estimate the incidence of patients consulting general practitioners (GPs) for a tick bite and to describe their characteristics, their management and the context of the bite.

**Method:**

We conducted a study using the French Sentinelles network, a national surveillance system that collects data from a sample of GPs. From May 2023 to April 2024, an indicator *‘Consultations with a tick bite’* was added to the routine data collection, in addition to the ongoing Lyme borreliosis surveillance. National and regional incidences were estimated using a hierarchical Bayesian model incorporating spatial and temporal dependencies to account for data sparsity. A tick collection was also carried out in order to identify the ticks and analyse the pathogens they might transmit.

**Results:**

A total of 179 GPs participated and reported 483 consultations for a tick bite episode. The estimated national incidence was 324 cases per 100,000 population (95% CI [290; 369]) over the study period, peaking in May-June 2023. Incidence was highest in the southwestern and northeastern France. Children represented one quarter of cases and nearly half of patients consulted in general practice without clinical symptoms. Of the 86 ticks analysed, 24% tested positive for at least one of the pathogens tested.

**Conclusion:**

These findings provide new insights into the epidemiology of tick bites in France and contribute to assessing population exposure to ticks, informing future prevention and public health strategies.

**Supplementary Information:**

The online version contains supplementary material available at 10.1186/s12889-026-27498-8.

## Introduction

Ticks are the primary vectors of human disease in the Northern hemisphere transmitting pathogens to humans through biting [1]. In mainland France, as in the northern hemisphere, the most common and well-known tick-borne disease (TBDs) is Lyme borreliosis (LB), caused by bacteria of the *Borrelia burgdorferi* sensu lato complex [1]. In Western Europe, including France, *Ixodes ricinus* has been identified as the main tick species vector [2]. In 2024, the incidence rate of LB cases seen in general practice in mainland France was estimated at 53 cases per 100,000 population [CI 95%: 43; 63] [3]. Estimations show a decreasing trend over the 2020–2023 period, after peaking in 2018. *Ixodes ricinus* is capable of transmitting other pathogens such as protozoa of the genus Babesia, which cause babesiosis, or tick-borne encephalitis (TBE) virus. Geographical distribution of ticks and epidemiology of TBDs are evolving, partly due to anthropogenic and climate changes. For instance, the Crimean-Congo hemorrhagic fever virus was detected for the first time in France in 2023 in *Hyalomma marginatum* ticks, ticks now established in the South of France [4, 5]. To date, there is no active national surveillance system for pathogens in ticks in mainland France.

The emergence and spread of TBDs pose significant challenges and many uncertainties remain. In this context, collecting data to estimate exposure of general population to tick bites is essential to identify high-risk geographical areas. This could help targeting prevention and public awareness efforts more effectively, contributing to a better understanding of tick ecology, and ultimately improving our overall understanding of tick-related risks. While several local studies on tick bite epidemiology have been conducted in Europe [6–9], French national data are scarce [10, 11]. The citizen science program CiTIQUE has collected more than 100,000 bite reports since 2017, which proved to be good indicators of spatial heterogeneity of LB cases when incorporated in a model [12], but may have a limited representativeness inherent to its participatory method. According to the 2024 health barometer survey, a nationally cross-sectional telephone survey on health behaviors and perceptions, 5% of the French population had been bitten in the last twelve months [13]. Of these, 15% (95% CI [3.7; 17.6]) consulted a healthcare professional following the tick bite (*data not published*). Although a medical consultation is not strictly recommended after a simple tick bite, general practitioners (GPs) are among the front-line healthcare professionals in terms of prevention and care after a tick bite. Current recommendations for managing a tick bite include prompt removal of the tick, if still attached, followed by disinfection of the bite site. Patients are then advised to monitor the bite area for the development of erythema migrans or other clinical symptoms. Neither serological testing nor antibiotic prophylaxis is recommended, regardless of patient characteristics (including the general population, pregnant women, children, or immunocompromised individuals).

Measuring consultations for tick bites in general practice could enable us to obtain quantitative data from the field to improve prevention strategies [14]. It also quantifies the workload for GPs, offering insights into the impact on the healthcare system. Given the lack of epidemiological data on consultation related to tick bites in general practice in France, we set up a study, based on an existing sentinel network surveillance system. The main aim was to estimate the incidence of consultations in general practice related to tick bite and to describe the epidemiological profile of individuals seeking medical advice for this reason. Consultation in general practice for LB are already monitored in France and have been the subject of several publications [15–17]. Our approach aims at quantifying the healthcare burden, but also at gathering detailed information on the characteristics and context of tick bites in patients consulting in general practice. This information could help improving messages on prevention towards the at-risk populations.

## Methods

### Study population

We conducted a nationwide study involving GPs from the French Sentinel network (Réseau Sentinelles) [18]. This general practice-based network on surveillance and research is made of volunteer GPs throughout mainland France, collecting epidemiological data in real-time. They constitute a diversified sample of the national GPs in terms of age and type of activity [19], representing about 2% of all GPs practicing in private practice in mainland France. Sentinel GPs report the number of cases seen in consultation for ten monitored health indicators, on a weekly basis, via a secured website. We aimed to recruit 200 GPs for this study to ensure that the incidence of consultations could be estimated with a 95% confidence interval of ± 8% around point estimates, based on an expected number of three cases per GP per year. GPs from all regions were invited to take part, regardless of the risk of exposure to tick bites. We also ensured that the proportion of participating GPs in relation to all GPs practicing in private practice in each region was close between regions (around 0.4%). The study took place between May 2023 (week 18) to April 2024 (week 17).

### Data collected

We collected data using a specific questionnaire developed for the study (Supplementary appendix 1). The case definition was as follows: patients presenting in consultation following a tick bite, whether or not they have any symptoms associated with the tick bite, excluding consultation specifically for LB, which is already monitored separately. We define a “tick bite episode” as a single exposure event during which a patient is bitten by one or more ticks. If a patient experiences multiple exposure during the study period and seeks medical care for each, these are considered separate episodes. Patients for whom a tick bite could not be clearly identified were not included. Each week, Sentinel GPs participating in the study had to report the number of cases they had seen in consultation (or teleconsultation) following a tick bite (excluding LB) in mainland France between May 2023 and April 2024, in addition to those monitored routinely. Besides demographic data and tick bite time and geographical location, they also had to report data on the activities while being bitten, the reason for consultation, potential clinical signs, history of tick bites and clinical management. A data quality control phase was carried out to correct discrepancies or fill in missing information. If needed, GPs could be contacted to obtain missing information or to correct inconsistent information.

### Tick identification, description and analysis

Sentinel GPs received materials for tick collection. If the patient came in consultation with the tick attached to the skin (or brought it with him/her), the GP could send it for identification and analysis as part of the study. The ticks included in the study were exclusively those spontaneously brought in by patients as part of their medical consultation. Ticks removed, stored in a tube containing 70% ethanol, were sent to our study laboratories by mail for identification and description. Tick species, stage, gender and degree of engorgement were determined after microscopic examination using identification keys [20, 21]. Ticks were determined as engorged when body modification characteristics of engorgement were observed, which appear after more than 24 h of feeding [22, 23]. All ticks received in 2023 were tested for the presence of tick-borne pathogens. This choice was based on practical considerations rather than random sampling, as too few specimens were collected in 2024 (*n* = 14) to perform further analyses. Tick DNA was extracted using the DNA insect kit (Macherey Nagel, Germany) and analyzed by microfluidic chips (Standard Biotools, USA). Microorganisms detected were validated using specific PCR and sequencing [24, 25]. Individual results were not sent back to the patients or to the GPs.

### Statistical analysis

Weekly departmental (NUTS-3) incidence rates per 100,000 population $\:{i}_{k,w}^{100}$ (week = *w*, department = *k*) were estimated using a spatio-temporal hierarchical Bayesian framework as described in Knorr-Held [26]. This model allows spatio-temporal smoothing of data when information is sparse.

GPs reported the number of tick-bite cases irregularly in time. First, we transformed these reports into regular weekly information as described in the Sentinelles protocol [27], yielding case counts $\:{\stackrel{\sim}{X}}_{k,w,gp}$ for GP *gp* in week *w* and department *k*.

We linked incidence rate to the case counts $\:{\stackrel{\sim}{X}}_{k,w,gp}$ using:

$${\stackrel{\sim}{X}}_{k,w,gp}\:\sim\:Poisson\left({i}_{k,w}^{100}\frac{{Pop}_{k}}{{M}_{k}\mathrm{*}100000}\right)$$

Where $\:{Pop}_{k}$ the department population and $\:{M}_{k}$ the total number of GPs in department *k*.

To estimate a smooth incidence over space and time, we assumed the following additive structure for log-incidence:

$$\mathrm{log}\left({i}_{k,w}^{100}\right)=\:{\phi\:}_{k}+{\gamma\:}_{w}+{\delta\:}_{k,w}$$

Where $\:{\phi\:}_{k}$ is a spatially structured effect modeled via an Intrinsic Conditional Autoregressive (ICAR) prior based on a geographical adjacency matrix (Supplementary appendix 2) and with standard deviation $\:{\sigma\:}_{space}$; $\:{\gamma\:}_{w}$ is a temporally structured effect modeled as a second-order random walk with standard deviation $\:{\sigma\:}_{time}$; and $\:{\delta\:}_{k,w}$ is an unstructured interaction term modeled as an i.i.d. Gaussian noise with zero mean and standard deviation $\:{\sigma\:}_{interaction}$.

Penalized Complexity (PC) priors were assigned to $\:{\sigma\:}_{space}$, $\:{\sigma\:}_{time}$ and $\:{\sigma\:}_{interaction},$ with hyper parameters (1, 0.01), (1, 0.01) and (0.5, 0.01) respectively. The national incidence in week *w* was computed by weighting each department by its population $\:{i}_{w}^{100}=\sum\:{k}po{p}_{k}\:{i}_{k,w}^{100}/\sum\:{k}po{p}_{k}$.

We estimated the incidence using the xpoisson family in the R-INLA package.

We estimated a time-changing age distribution of cases at the national level, according to three age classes (0–14, 15–64, 65 + years). We modelled $\:{D}_{t,a}$, the number of cases in age group *a*, as a multinomial distribution [28]:

$$\left({D}_{t,1},{D}_{t,2},{D}_{t,3}\right)\sim{Multinomial}\left({{\pi\:}_{Age}}_{t,1},{{\pi\:}_{Age}}_{t,2},{{\pi\:}_{Age}}_{t,3}\right)$$

Finally, the national age specific incidence rates were obtained as:

$${i}_{w,a}^{100}={{\pi\:}_{Age}}_{t,a}\mathrm{*}\frac{\sum\:_{a}po{p}_{a}}{{pop}_{a}}\mathrm{*}{i}_{w}^{100}$$

Where $\:{pop}_{a}$ is the national population in age group *a*.

We generated 1000 posterior samples of weekly departmental incidence rates, subsequently aggregated to regional (NUTS-2) and national levels, as well as to monthly and annual time scales, and stratified by age groups. Point estimates and 95% credible intervals were derived as the posterior mean and 2.5th and 97.5th percentiles.

Sensitivity analysis included changes to the prior hyper parameters, removal of the interaction term, and comparison with alternative likelihoods (Poisson, n binomial) using rounded integer data.

Case characteristics were described with numbers and percentages, as well as tick identification, description and pathogen detection. All analyses were performed using R software version 4.5 and INLA package (v 24.12.11).

## Results

### SGPs participation

A total of 179 Sentinel GPs were included in the study. Of these, 176 (98%) participated in at least one week of surveillance. Their median age was 46 [IQR = 39; 60] and 50% were women (*n* = 90) (*data not shown*). On average, 122 Sentinel GPs (min: 97; max: 141) contributed to the surveillance every week over the entire study period. The average weekly number of GPs participating in surveillance is shown in Supplementary appendix 3 and ranged from 0 to 2.7. Overall, 29 departments (NUTS 3) were not covered by any Sentinel doctors, including all departments in the Normandy and Poitou-Charentes regions.

During the study period, 131 (73%) Sentinel GPs reported having seen at least one patient in a consultation related to a tick bite. A total of 483 cases of patients seen in a consultation related to a tick bite were included in the study. Geographical distribution of participating Sentinel GPs according to the number of cases reported are detailed in Supplementary appendix 4.

### Incidence estimations

**Fig. 1:**
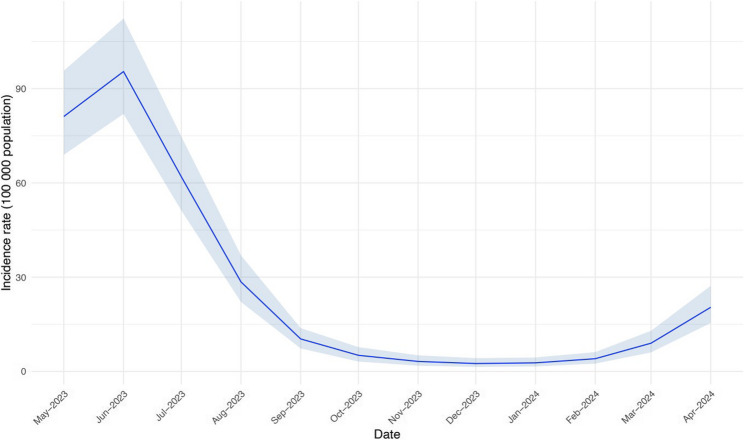
Evolution of monthly incidence rates of consultation following a tick bite, France, 2023–2024

**Fig. 2:**
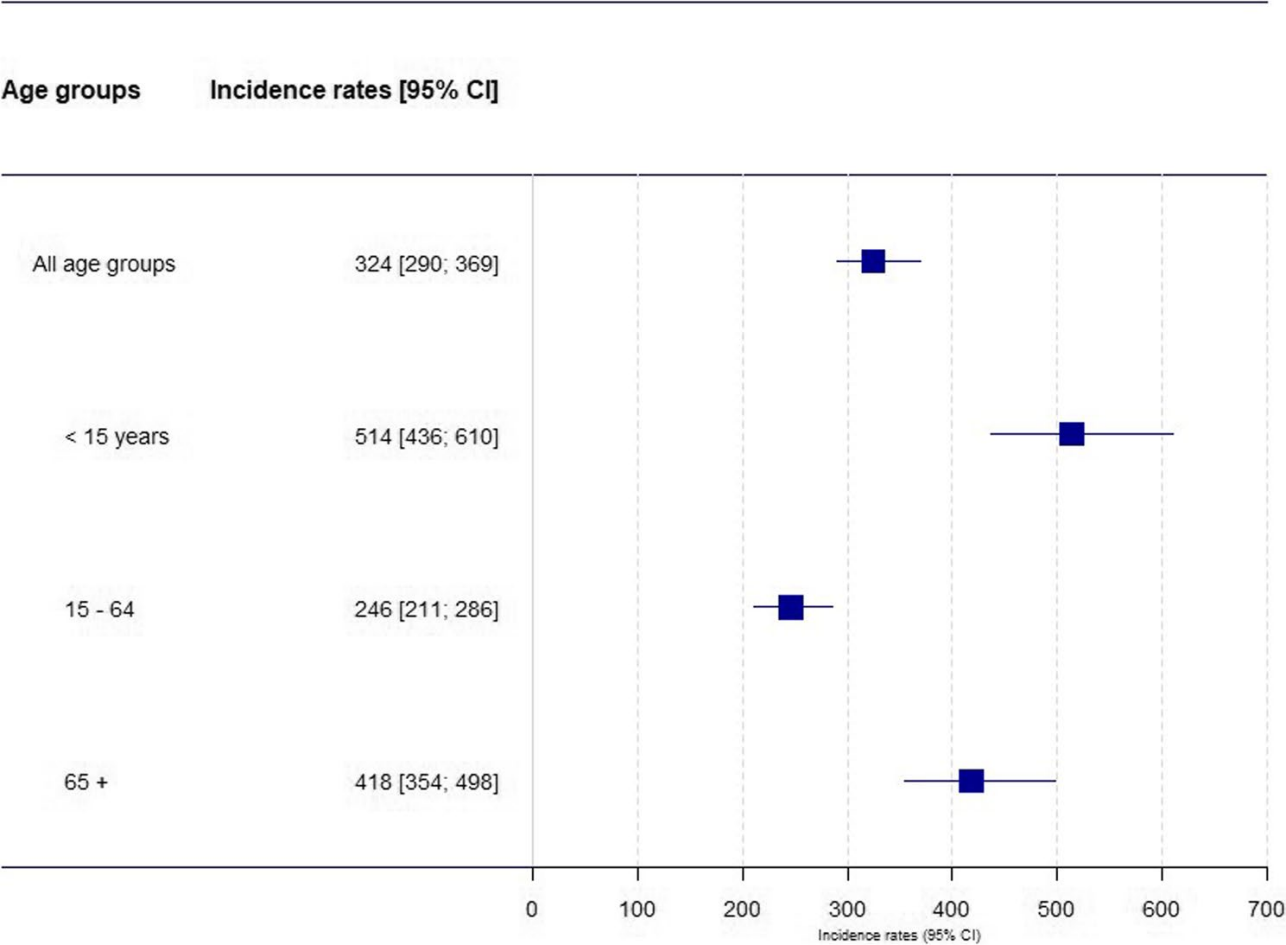
Incidence rates of consultation following a tick bite, by age groups, France, 2023–2024

**Fig. 3:**
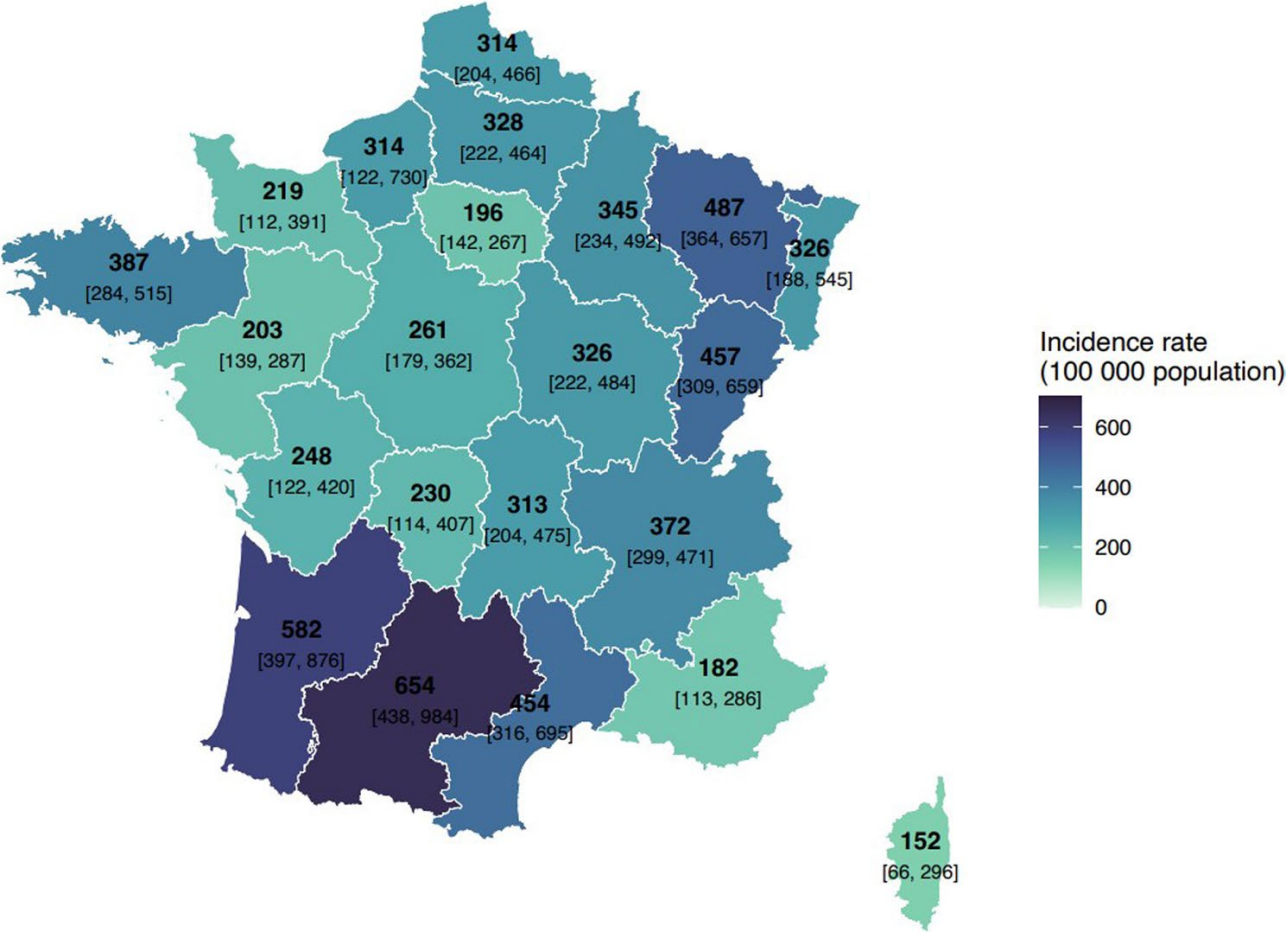
Incidence rates of consultation following a tick bite, by regions, France, 2023–2024

Between May 2023 and April 2024, the incidence of patients consulting in general practice following a tick bite episode in mainland France was estimated at 215,712 cases [192,796; 245,116], corresponding to an annual incidence rate of 324 per 100,000 population (95% CI [290; 369]). The evolution of the monthly incidence rates is shown in Fig. 1. The highest monthly incidence rates were recorded in May, June and July 2023 with 81 [69; 96], 95 [82; 112] and 62 [51; 75] cases per 100,000 population respectively. Incidence rates then declined between August and October 2023, reaching a very low level during the winter. They rose again by March 2024. The annual incidence rate of patients seen in consultation related to a tick bite was significantly higher in the 0–14 age group, compared to 15–64 age group (514 [436; 610] cases vs. 246 [211; 286] cases per 100,000 population) (Fig. 2). Tick bites consultations were reported across all regions, though substantial regional variations were observed (Fig. 3). Estimated annual incidence rates ranged from 152 [66; 296] cases per 100,000 in Corsica to over 500 per 100,000 population in several southwestern regions.

### Description of case characteristics

Cases characteristics are presented in Table 1. The median age of patients seen in consultation following a tick bite was 45 years-old [IQR = 16; 64] and 55% (*n* = 261) were women. About half of them (*n* = 243) consulted for medical advice regarding the tick-bite (in the absence of clinical signs), 18% (*n* = 94) for clinical advice due to symptoms and 16% (*n* = 144) for tick removal. Most patients (87%; *n* = 416) had been bitten by a single tick and 10% (*n* = 48) by two ticks during this episode. Tick bites were most frequently located on lower limbs (33.5%; *n* = 173) and trunk (19%; *n* = 99). In children (< 15 years-old), the head and the neck were the most common sites (42%). The presumed activity associated with the tick bite was leisure in 90% (*n* = 428) and work or school in 4.5% (*n* = 25). After examination by the GP, almost a third of patients (*n* = 128) had clinical signs that could be associated, with the tick bite. In 79% of cases (*n* = 101), it was an inflammation at the site of the bite. Whether symptomatic or not, about 18% (*n* = 81) of the patients were prescribed antibiotic therapy and 9% (*n* = 36) serological testing for LB by the GP. Concerning history of exposure to tick bites, about 19% (*n* = 80) of patients were previously bitten in the last 12 months and 4.5% (*n* = 20) of patients had already had a tick-borne disease in the past.

**Table 1:** Description of cases seen in general practice consultation following a tick bite episode, mainland France, May 2023-April 2024 (*N* = 483)

Case description	Consultation following a tick bite episode (LB excluded) *N* = 483	
	*n*	%
**Patient characteristics**		
Age (median in years) [IQR]	45 [16; 64]	
Age (in years) *(m.d.=14)*		
0–4	32	6.8
5–9	54	11.5
10–14	25	5.4
15–34	69	14.7
35–54	115	24.5
55 or more	174	37.1
Gender *(m.d.=9)*		
Woman	261	55.1
Men	213	44.9
**Information on the tick bite**		
Reason(s) for consultation (*multiple answers possible)* *(m.d.=2)*		
For medical advice following a tick bite in the absence of clinical signs	243	45.9
For medical advice following clinical signs	94	17.8
To remove tick(s) or rostrum residues	144	27.2
On the advice of a healthcare professional (nurse, pharmacist, …)	23	4.3
Other reason	25	4.7
Number of tick bite reported during the same episode *(m.d.=3)*		
1	416	86.7
2	48	10.0
3 or more	16	3.3
Tick bite location on the body *(multiple answers possible) * *(m.d.=4)*		
Lower limbs	173	33.5
Trunk	99	19.2
Upper limbs	61	11.8
Head	51	9.9
Inguinal region	44	8.5
Back	40	7.8
Neck	34	6.6
Axillary region	14	2.7
Presence of a tick during consultation *(m.d.=0)*		
Yes, full	108	22.4
Yes, partial	58	12.0
No	317	65.6
**Background to the tick bite**		
Probable duration of attachment *(m.d.=55)*		
Less than 12 h	168	39.3
Between 12 and 24 h	131	30.6
Between 24 and 48 h	68	15.9
48 h or more	61	14.3
Presumed activity at time of tick bite *(multiple answers possible) * *(m.d.=13)*		
Work/school activity	23	4.8
Leisure activities (walking, sports, picnics, gardening, etc.)	428	90.1
Hunting, fishing	5	1.1
Other	19	4.0
**Clinical manifestations**		
Presence of clinical signs associated, according to the GP, with the tick bite *(m.d.=29)*		
Yes	128	28.2
No	326	71.8
If yes, details of symptoms (*multiple answers possible*) *(m.d.=0)*		
Cutaneous inflammation at the bite site	101	78.9
Myalgias and/or arthralgias	5	3.9
Headache	5	3.9
Ulcer or eschar at the bite site	5	3.9
Fever or feeling of fever	4	3.1
Other	23	17.0
**Medical care**		
Prescription of antibiotic therapy *(m.d.=22)*		
Yes	81	17.6
No	380	82.4
Prescription of serological test (*multiple answers possible*) *(m.d.=102)*		
Yes, serology for Lyme borreliosis	36	9.4
Yes, serology for tick-borne encephalitis^‡^	1	0.3
No	345	90.3
**History of exposure to tick bites**		
In the last 12 months, number of tick bite episodes reported by patient *(m.d.=53)*		
None	350	81.4
1	35	8.1
2 or more	45	10.5
Over the past 12 months, number of previous consultations related to a tick bite *(m.d.=53)*		
None	407	94.7
1 or more	23	5.3
History of tick-borne disease *(m.d.=42)*		
Yes	20	4.5
No	421	95.5
If yes, which one *(m.d.=0)*		
Lyme borreliosis	18	90.0
Tick-borne encephalitis	0	0.0
Rickettsiosis	1	5.0
Other without any precision	1	5.0

*m.d. *missing data

### Description of ticks and microbiological results

During the study period, 100 ticks were sent in by GPs for identification and description (Table 2). The majority were *Ixodes ricinus* species (88%; *n* = 88), four *Dermacentor marginatus*, three *Dermacentor reticulatus*, three *Rhipicephalus sanguineus* s.l. and two *Hyalomma marginatum*. The last three species were found on patients bitten in departments in southeastern France. About two thirds were nymphs (67%; *n* = 66) and 60% (*n* = 56) were engorged, meaning that they were removed more than 24 h after biting. All ticks collected in 2023 (86/100) were screened to detect microorganisms. In total, 77 *I. ricinus*, 3 *R. sanguineus* s.l., 3 *D. marginatus*; 2 *D. reticulatus* and 1 *H. marginatum* were analyzed. Of them, 24% (*n* = 21) were positive for at least one pathogen tested: 9% (*n* = 8) were positive *for Borrelia* spp., 12% (*n* = 10) for *Rickettsia* spp. and 4% (*n* = 3) for *Babesia venatorum*. The results by tick species are detailed in Supplementary appendix 5.

**Table 2:** Identification, description and results of microorganism detection of ticks, mainland France, May 2023-April 2024

	*n*	%
Species (*n* = 100)		
* Ixodes ricinus*	88	88.0
* Dermacentor marginatus*	4	4.0
* Rhipicephalus sanguineus* s.l.	3	3.0
* Dermacentor reticulatus*	3	3.0
* Hyalomma marginatum*	2	2.0
Developmental stage (*n* = 98)		
* Larvae*	1	1.0
Nymph*	66	67.3
* Adult*	31	31.6
* Female*	*27*	
* Male*	*4*	
Engorgement (*n* = 93)		
* Unengorged (flat tick)*	37	39.8
* Engorged*	56	60.2
Detected DNA sequences of ticks collected in 2023 (*n* = 86)		
Positive for at least one microorganism**	21	24.4
* Borrelia* spp.	8	8.9
* B. afzelii*	*3*	
* B. valaisiana*	*2*	
* B. burgdorferi* sensu stricto	*1*	
* B. spielmanii*	*1*	
* B. miyamotoi*	*1*	
* Rickettsia* spp.	10	11.6
* R. helvetica*	*8*	
* R. massiliae*	*1*	
* R. raoultii*	*1*	
* Babesia venatorum*	*3*	3.5
* Neoehrlichia mikurensis*	2	2.3
* Anaplasma phagocytophilum*	1	1.3

*including 3 Dermacentor marginatus nymphs,**including 3 co-infections

## Discussion

### Main findings

This study provides an estimation of the incidence of tick bite-related consultations in general practice in mainland France. Most bites were reported between May and August, and in the south-west and north-east regions. About one quarter of these consultations following a tick bite concerned children under 15 years-old. Almost half of tick bite-related consultations were for seeking medical advice in the absence of clinical signs.

### Interpretation and comparison with other studies

Our study highlights the high level of tick bite-related consultations (excluding LB), which are estimated to be five times higher than Lyme borreliosis consultations (incidence rate estimated at 53 [43; 63] cases for 100,000 population in 2024). The incidence rate of consultations following a tick bite episode is higher in children than in adults, which contrasts with the age distribution of patients consulting for Lyme borreliosis [16], but aligns with data from the CiTIQUE programme [29]. This difference could be attributed to a higher risk of tick bites for children but also to a higher risk perception by parents, being more inclined to consult a healthcare professional for the removal of a tick or for medical advice for their child than for adults. Most studies on tick bites focus on adults because of limitations from their methods of investigation [30, 31] while tick bites on children remain understudied. The seasonal distribution of tick bites throughout the year is consistent with previous findings [29, 32, 33]. This period (between May and August) corresponds to the ticks’ period of highest activity and public exposure, which has been well described elsewhere [34].

Patients consulting GPs for tick bites were reported across all regions of mainland France. However, we observed notable geographical disparities, with the highest incidence rates in southwestern and northeastern France, and the lowest in the north and south-east. These results are consistent with the geographical distribution of the 2024 Nationally cross-sectional survey on health behaviors and perceptions, where the regions with the highest proportion of people bitten in the last 12 months were also the north-east and south-west of France [30]. However, these differences between regions should be interpreted carefully, because of the large number of factors involved and the lack of precision of estimations in certain regions due to the small number of GPs participating in the study (see Supplementary appendix 6). This regional distribution of the incidence of patients seen in consultation following a tick bite episode appears to differ in some parts of the country from the regional distribution of Lyme borreliosis incidence. Several factors need to be considered, as the relationship between tick-bite exposure and infection is complex [34, 35] as well as risk perception [36]. Among these factors, tick species distribution varies between regions, and therefore the proportion of *Ixodes ricinus* transmitting the bacteria responsible for Lyme borreliosis [10]. As our study was conducted in general practice, we can assume that our results do not necessarily reflect the incidence of tick bites in the general population. Indeed, people knowledge on preventive measures including how to remove an attached tick and their behaviour in seeking care may vary according to their socio-demographic and occupational characteristics as well as their geographical and individual level of perceived exposure [37, 38]. Given the difficulty to establish the relation between density of ticks, risk of tick bites and risk of TBDs, wider studies are needed to specifically explore the frequency of outdoor activities and preventive measures, but also seeking care practices (e.g. self-checking for symptoms, visit to a local pharmacy, etc.).

In our study, half of all patients came to the GP following the tick-bite in the absence of symptoms. Comparison with data from the 2019 health barometer survey shows that the proportion of patients seeking medical advice in the absence of clinical signs was similar (44%) and 19% consulted for tick removal (compared to 25% in our study) [37]. Based on the estimated incidence following a tick bite episode in our study, this means that more than 100,000 individuals consult a GP for medical advice in the absence of clinical signs and around 60,000 for tick removal per year. This result raises the question of consultations that could be avoided by reinforcing prevention messages and increasing public awareness on tick bites and especially how to remove an attached tick [39]. Furthermore, it is recommended to remove a tick from the skin as soon as possible after the exposure to limit the risk of TDB’s transmission, at best before 24 h of attachment, which is not always compatible with the delay to consult a GP. It is therefore not recommended to consult a GP for tick removal, and better to increase knowledge on how to remove a tick by oneself. According to the Nationally cross-sectional surveys on health behaviors and perceptions, the proportion of people who felt well informed about LB increased 29% to 42% between 2016 and 2019 among people feeling exposed to tick bites. This figure has been remained stable at 40% between 2019 and 2024 [13, 37]. This result is encouraging but does not necessarily imply that people are correctly applying protective measures.

Regarding microorganism detection, the proportion of ticks infected by *Borrelia* spp. collected in our study was lower than in ticks collected in the CiTIQUE program: among the 2009 biting ticks collected between 2017 and 2019 that CiTIQUE analysed, 15% were infected by *Borrelia* vs. 9% in our study [40]. However, the results were very similar for the detection rate of other microorganism (about 15%). This difference may be due to the small sample of ticks we analysed (86 ticks), to the years and to the origin of biting ticks sampled. Comparison of the proportion of infected ticks to other studies in Europe is limited because of heterogeneity between countries [41]. Overall, the proportion of *Borrelia*-infected ticks collected from humans ranged from 6% in Italy (2017–2019) to 29% in the Netherlands (in 2013) [32, 42, 43]. In France, to date, only local data on the prevalence of *Borrelia* infection in ticks have been published and vary from 9% to 24% [44–46].

### Limits and strengths

The main limitation of our study is the low number of GPs participating in some regions. This issue was addressed through the use of a Bayesian spatio-temporal model. This model provides a robust framework for estimating incidences and allows for missing data to be managed while considering spatial and temporal correlations and quantifying the associated uncertainty (Supplementary appendix 7). Another potential limitation concerns the representativeness of participating Sentinelles GPs. Additional analyses (data not shown) indicated that the average number of LB cases reported annually was significantly higher among participating GPs than among non-participants. This may suggest that Sentinelles GPs involved in the study were more aware and interested in TBDs, potentially leading to overestimation of incidence. Furthermore, selection bias within regions—for instance, differences between urban and rural practices—may partially explain regional disparities. For these reasons, inter-regional comparisons should be interpreted cautiously, considering the estimated confidence intervals.

Conversely, the main strength of our study lies in the high-quality data collected from a nationwide network of GPs. Leveraging an existing surveillance system allowed for standardized data collection following a unified protocol with with trained GPs. This study shows that continuous surveillance of tick bites in general practice could complement other surveillance systems of TBDs, especially adding information on tick bite exposure and on population knowledge about ticks and TBDs.

## Conclusion

This study contributes to expanding the data and knowledge available on tick bites in France. complementing other existing sources, through the prism of general practice consultations. Further studies are needed to better understand how and why patients are seeking care following a tick bite. Improving the diffusion of prevention messages to increase public awareness and knowledge about tick-bites, their removal and their associated risks is essential to avoid potentially unnecessary consultations with GPs and better address the problem of tick-borne diseases. These findings highlight the importance of primary care and healthcare provider awareness on tick bite management.

Having lasted just one year, our study does not allow evaluating temporal trends. Data compiled over several years would be needed to confirm national and regional trends. In a context of rising risks related to emerging diseases, continuous monitoring of tick bites will be necessary in coming years and this study can be considered as a pilot for a permanent monitoring indicator in general practice.

## Supplementary Information

Supplementary Material 1.

## Data Availability

The datasets used and/or analysed during the current study are available from the corresponding author upon reasonable request.
