# Supplementary material for: Consultations in general practice related to a tick bite episode in mainland France in 2023–2024
Source: BMC Public Health. 2026 Apr 29;26:1889. doi: 10.1186/s12889-026-27498-8 (PMC13274222; doi:10.1186/s12889-026-27498-8)
Supplement: Supplementary file 1 — Supplementary Material 1. [file 12889_2026_27498_MOESM1_ESM.docx]

**Supplementary appendix 1.** Case description form completed by the GP

# **Patient characteristics and reason for consultation**

- Age (in years) or age unknown
- Gender: < Male / Female / Not specified >

# **Information about the tick bite(s)**

- Reason(s) for consultation (*multiple answers possible*): < For medical advice following a tick bite in the absence of clinical signs / To remove one or more ticks / To remove rostrum residue / For medical advice following the onset of clinical symptoms / On the advice of a healthcare professional (e.g. nurse, pharmacist) / For medical advice following laboratory results / Other reason
- Number of bites reported by the patient in connection with the current consultation: <-->
- Number of bites you observed during the consultation: <-->
- Location of tick bite(s) observed during today's consultation, or reported by the patient in connection with this consultation (multiple answers possible): < Head / Neck / Torso / Back / Axillary region / Upper limbs / Groin area / Lower limbs >
- During the consultation, did you observe one or more ticks (on the patient or brought in by the patient)? < Yes, whole / Yes, partial / No >

**[If yes]**

- Did you send the tick(s) for analysis as part of the study? < Yes / No >

# **Context of the tick bite(s)**

- Date of the bite: DD/MM/YYYY

*Note: If you do not know the exact day of the bite, you can enter an approximate date or just the month and year.*

- Probable duration of attachment of the tick(s): < Less than 12 hours / Between 12 and 24 hours / Between 24 and 48 hours / Between 24 and 48 hours / 48 hours or more / Unknown >
- Location of the bite: < Abroad / France / Unknown >

**[If France]** Department number < Free text >

- Presumed activity at the time of the bite (multiple answers possible): < Work/school activity / Leisure activity (walking, picnicking, gardening, sport, etc.) / Hunting/fishing / Other >

# **Description of clinical symptoms likely ot be related to one or more tick bites**

- Did the patient show clinical signs likely related to the tick bite(s)? < Yes / No / Unknown >

*Reminder: For erythema migrans or clinical signs of Lyme disease, report it in the ‘Lyme disease’ indicator.*

**[If yes]** Date of onset of symptoms: < Date >

**[If yes]** Symptoms (multiple answers possible): < Fever or feeling feverish / Muscle and/or joint pain / Vomiting / Headache / Inflammation at the bite site / Ulcer or bedsore at the bite site / Other >

**[If Other]** Specify: < Free text >

# **History of exposure to tick bites**

- Over the past 12 months, number of episodes of stings reported by the patient: <-->

*Note: Do not count the current episode.*

- Over the past 12 months, number of consultations related to these bite incidents: <-->

*Note : Si le patient a consulté plusieurs fois un médecin (vous ou un autre médecin) pour un même épisode, ne retenir qu’une seule consultation.*

- Does the patient have a history of tick-borne disease? < Yes / No / Unknown >

**[If yes]** Which one? < Lyme disease / Tick-borne encephalitis / Rickettsiosis / Other >

# **Description of care**

- Prescription of antibiotic therapy: < Yes / No / Unknown >

## Prescription of serology (*multiple answers possible*): < Yes, serological test for Lyme disease / Yes, serological test for tick-borne encephalitis (TBE) / No >

**Free comment < Free text >**

*If you wish, you can provide any useful details about this case below. Please be careful not to include any sensitive information (health data related to another diagnosis) or information that could identify the patient.*

**
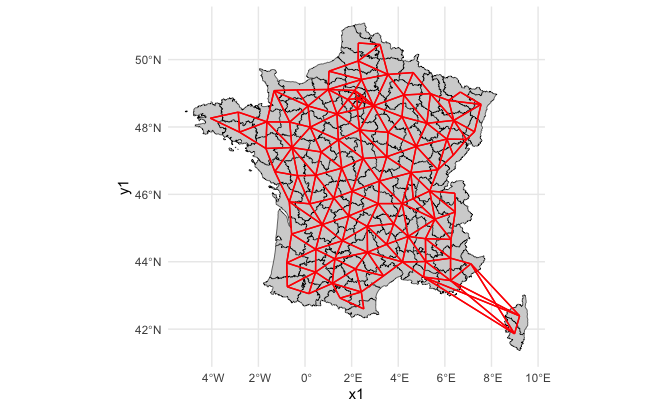
**

**Supplementary appendix 2**. Geographical adjacency map of the French mainland departments


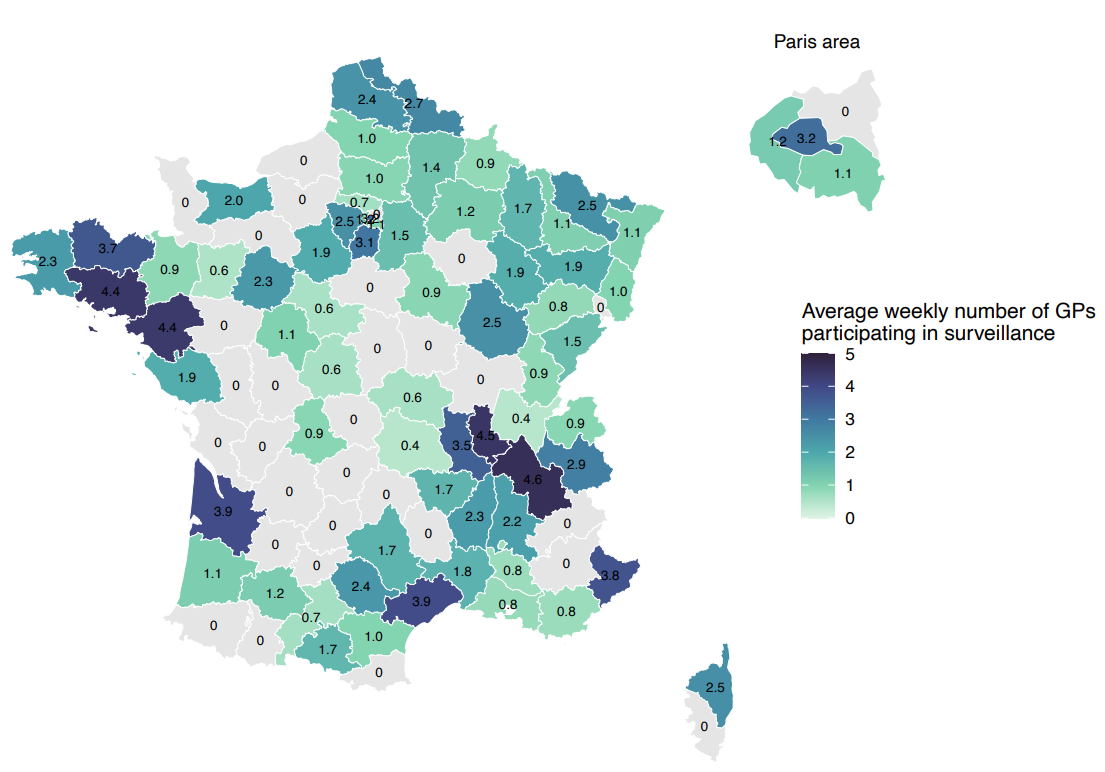


**Supplementary appendix 3**. Average weekly number of GPs participating in *Sentinelles* surveillance, by departments, mainland France, May 2023-April 2024


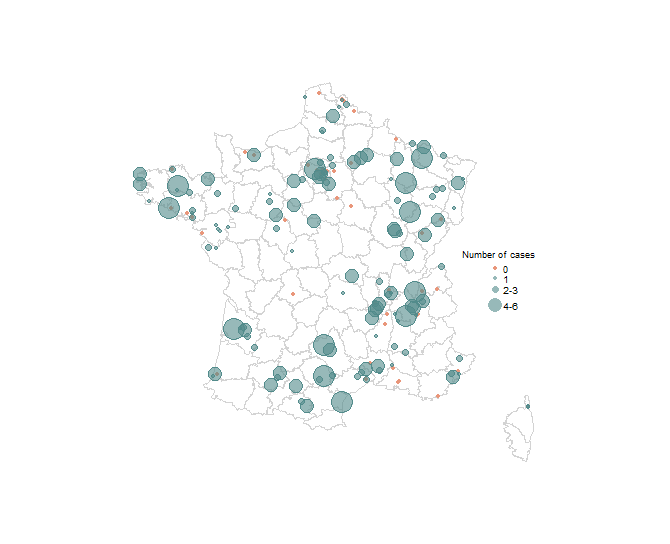


**Supplementary appendix 4.** Geographical distribution of participating Sentinelles GPs and reported tick bite-related consultations, mainland France, 2023–2024.

*Circles represent the towns where Sentinelles GPs participating are based. The size of each circle indicates the number of tick bite-related consultations reported. Red dots indicate GPs who did not report any cases during the study period. When multiple GPs from the same town participated, values correspond to the average number of consultations reported.*

| **Supplementary appendix 5.** Detected DNA sequences by tick species, mainland France, 2023-2024 (n=86) | | | | | | |
| --- | --- | --- | --- | --- | --- | --- |
|  | **Total  (N=86)** | **By tick species** | | | | |
|  |  | *Ixodes ricinus N=77* | *Rhipicephalus sanguineus s.l. N=3* | *Dermacentor marginatus N=3* | *Dermacentor reticulatus N=2* | *Hyalomma marginatum N=1* |
| **Detected**  **DNA sequences** |  |  |  |  |  |  |
| Positive for at least one pathogen*** | 21 (24.4%) | **19^¥^** | **1** | **0** | **1** | **0** |
| *Borrelia* spp. | *8 (8.9%)* | **8** |  |  |  |  |
| *B. afzelii* | *3* | 3 |  |  |  |  |
| *B. valaisiana* | *2* | 2 |  |  |  |  |
| *B. burgdorferi* sensu stricto | *1* | 1 |  |  |  |  |
| *B. spielmanii* | *1* | 1 |  |  |  |  |
| *B. miyamotoi* | *1* | 1 |  |  |  |  |
| *Rickettsia* spp . | *10 (11.6%)* |  |  |  |  |  |
| *R. helvetica* | *8* | **8** |  |  |  |  |
| *R. massiliae* | *1* |  | 1 |  |  |  |
| *R. raoultii* | *1* |  |  |  | 1 |  |
| *Babesia venatorum* | *3 (3.5%)* | **3** |  |  |  |  |
| *Neoehrlichia* | *2 (2.3%)* | **2** |  |  |  |  |
| *Anaplasma phagocytophilum* | *1 (1.3)* | **1** |  |  |  |  |
| ¥ 3 co-infections: 1 co-infection *B. afzelii* and *R. helvetica*, 1 co-infection *B. afzelii* and *Neoehrlichia and 1 co-infection* B. spielmanii and *Babesia venatorum* | | | | | | |

**
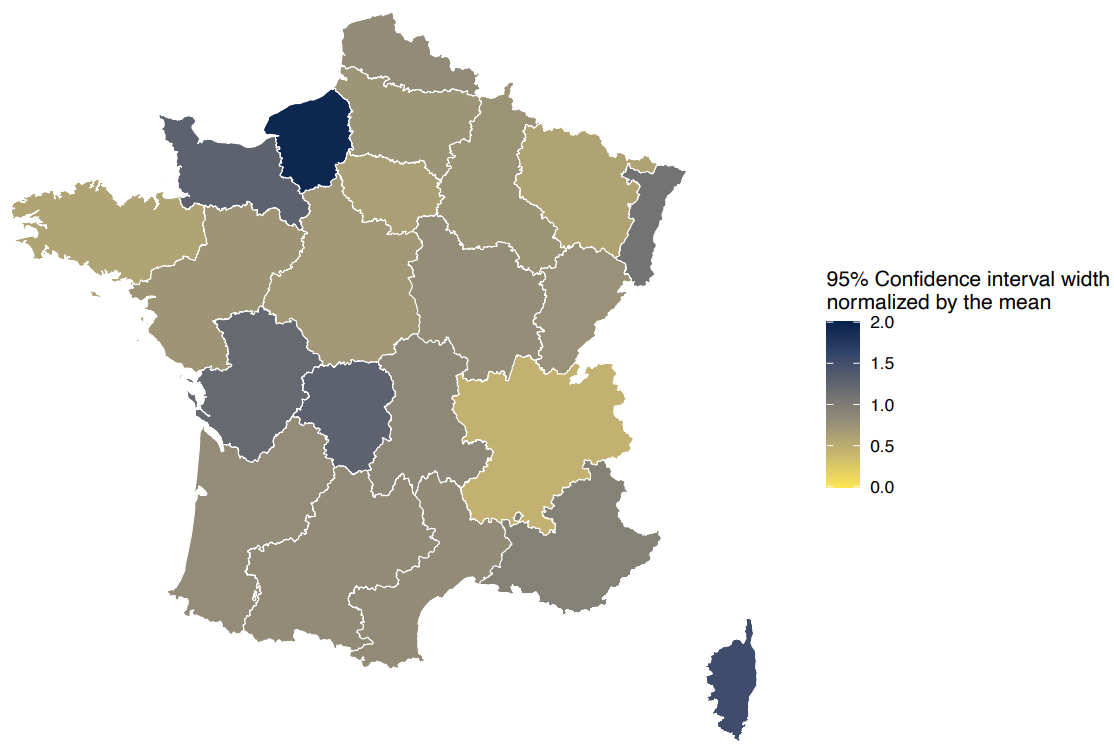
**

**Supplementary appendix 6**. Distribution of 95% confidence intervals width normalized by the mean, by regions, mainland France, May 2023-April 2024

**Supplementary appendix 7**. Sensitivity analysis of estimated incidence rates per 100,000 inhabitants (95% CI), mainland France, May 2023-April 2024

| **Age** | **Main analysis** | **Poisson likelihood** | **Nbinomial**  **likehood** | **Without interaction** | **HP* incidence (10,0.1)** | **HP* incidence (0.1,0.01)** | **HP* age (10,0.1)** | **HP* age (0.01,0.01)** |
| --- | --- | --- | --- | --- | --- | --- | --- | --- |
| **Total** | 324 [290 ; 369] | 334 [300 ; 378] | 330 [294 ; 377] | 323 [288 ; 363] | 326 [288 ; 369] | 319 [288; 353] | 324 [290 ; 369] | 324 [290 ; 369] |
| **[0,15)** | 514 [436 ; 610] | 518 [435 ; 619] | 514 [428 ; 621] | 501 [422 ; 596] | 506 [417 ; 610] | 499 [426 ; 581] | 506 [425 ; 598] | 544 [465 ; 642] |
| **[15,65)** | 246 [211 ; 286] | 257 [221 ; 299] | 252 [216 ; 296] | 247 [212 ; 287] | 248 [216 ; 290] | 243 [211 ; 278] | 247 [213 ; 285] | 234 [202 ; 274] |
| **65+** | 418 [354 ; 498] | 430 [359 ; 505] | 426 [360 ; 507] | 417 [350 ; 496] | 421 [349 ; 499] | 414 [353 ; 480] | 419 [345 ; 493] | 426 [360 ; 497] |
